# Modulation of the electrical double layer in metals and conducting polymers

**DOI:** 10.1038/s41598-021-03948-8

**Published:** 2022-01-10

**Authors:** Jorge Morgado

**Affiliations:** Instituto de Telecomunicações and Department of Bioengineering, Instituto Superior Técnico, Universidade de Lisboa, Av. Rovisco Pais, 1049-001 Lisbon, Portugal

**Keywords:** Chemistry, Physics

## Abstract

The electrical double layer (EDL) formed at the interface between various materials and an electrolyte has been studied for a long time. In particular, the EDL formed at metal/electrolyte interfaces is central in electrochemistry, with a plethora of applications ranging from corrosion to batteries to sensors. The discovery of highly conductive conjugated polymers has opened a new area of electronics, involving solution-based or solution-interfaced devices, and in particular in bioelectronics, namely for use in deep-brain stimulation electrodes and devices to measure and condition cells activity, as these materials offer new opportunities to interface cells and living tissues. Here, it is shown that the potential associated to the double layer formed at the interface between either metals or conducting polymers and electrolytes is modified by the application of an electric field along the conductive substrate. The EDL acts as a transducer of the electric field applied to the conductive substrate. This observation has profound implications in the modelling and operation of devices relying on interfaces between conductive materials (metals and conjugated polymers) and electrolytes, which encompasses various application fields ranging from medicine to electronics.

## Introduction

The study of the electrical double layer (EDL) dates back to the nineteen century^1,2^. It is a scientific and technologically relevant topic as it is present in various areas, namely batteries, corrosion studies, sensors and actuators and in devices with interfaces. The most relevant situations, and those that are better understood and characterised, involve interfaces between metallic systems and electrolytes. With the discovery of the conjugated polymers^3^ and our ability to tune their conductivity, several applications have been developed, namely those involving their interfaces with electrolytes as an active component^4–16^. The EDL formed at the interface between such highly conductive systems and electrolytes is anticipated to share similarities with the metal/electrolyte situation. Here, a study on the modulation of the EDL formed at the interface between a liquid electrolyte [KCl solution and PBS (phosphate-buffered saline)] and conductive substrates, namely gold and the well-known highly conjugated polymer poly(3,4-ethylenedioxythiophene) doped with polystyrene sulfonic acid (PEDOT:PSS) is presented.

This study was motivated by our recent works on the application of electric fields to PEDOT:PSS films supporting neural stem cells culture to influence their differentiation^14–16^. In particular, we found that the application of an electric field along the PEDOT:PSS substrate influences positively the differentiation of the neural stem cells into neurons. It has not been clear how the field applied to the substrate influences the cell culture. It should be noted that similar reported studies on electrical stimulation involve setups with electrical stimulation applied directly to the cell culture medium(electrolyte)^17^. The findings reported in this letter may provide an explanation for the efficiency of our setup to influence the neural stem cells, as, by virtue of the EDL, there is an electrical field that extends into the bulk of the cell medium (electrolyte) away from the PEDOT:PSS surface. It remains to be discussed how far this field progresses into the cell culture medium but, despite the coating of PEDOT:PSS with poly-L-ornithine and laminin to favour cell adhesion, it is likely at origin of the electrical stimulation of the cells cultured on top.

It is found that the application of a bias to the PEDOT:PSS film in contact with an electrolyte (KCl(aq) or PBS) modifies the EDL potential as measured by the open circuit potentiometry (OCP) method. The effect is also found in gold/KCl(aq) and graphite (HOPG) /KCl(aq) interfaces. The EDL modification scales with the bias potential applied across the film under the electrolyte and should be present in the devices and setups involving conductive substrates/electrolyte interfaces where a bias is applied along the substrates. Two main examples are the electrolyte-gated electrochemical transistors and deep-brain stimulation electrodes, encompassing also sensors operating with such active interface.

## Results and discussion

**Figure 1:**
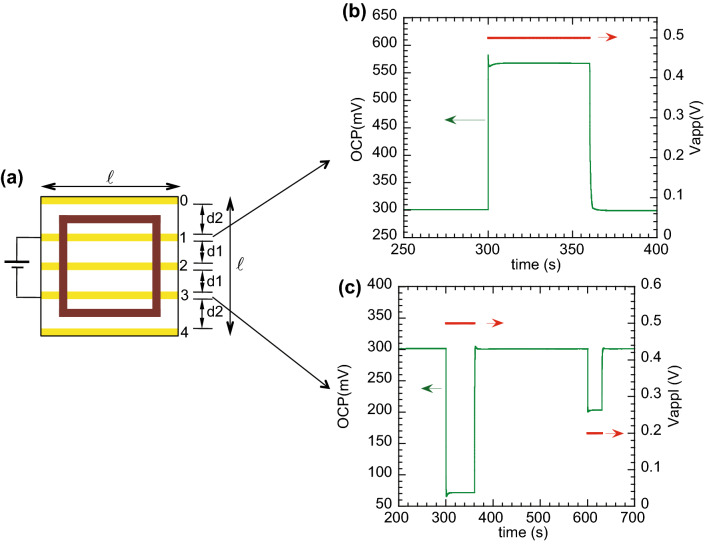
(**a**) Structure of a cross-linked PEDOT:PSS-based setup, consisting on three probing ITO stripes (ca. 1.49 mm width), ca. 2.75 mm apart, underneath the PEDOT:PSS film. Also shown is the electrolyte cubic container (lateral outside dimension of 1.7 cm, 1 cm high and wall thickness of about 2 mm prepared by 3D printing), which was glued on the PEDOT:PSS film at a nearly centred position. The Ag/AgCl reference electrode and the counter Pt electrode were immersed in the electrolyte, the bias leads were contacting the ITO stripes 1 and 3, while the probe lead (working electrode) contacted position 1, 2 or 3. (**b**) Variation of the EDL potential at position 1 when a + 0.5 V pulse (shown in red) is applied between contacts 1 and 3; (**c**) Variation of the EDL potential at position 3 when a + 0.5 V pulse followed by a + 0.2 V pulse are applied between contacts 1 and 3. The decrease of the EDL potential under a bias of + 0.2 V to 0.1 V evidences the scaling of the EDL potential change with the bias potential.

Figure 1a shows a typical setup used in the study of the EDL formed between PEDOT:PSS and an aqueous electrolyte. The PEDOT:PSS film was cross-linked to ensure its integrity upon contact with the aqueous solution^15^. The potential of the electrical double layer (EDL) was measured using open circuit potentiometry (OCP) in a three-electrode configuration, using an Ag/AgCl reference electrode and a platinum wire as the counter electrode. The ITO stripe is used as the working electrode. The selection of this geometry is due to the fact that ITO has a higher conductivity (3300 S/cm) than the PEDOT:PPS film (5 S/cm), thereby allowing a spatial localization for the electrical double layer potential assessment. This set up is similar to the one we have been using in the study of electrical stimulation of neural stem cells^14–16^.

OCP measurements were carried out as a function of time at room temperature, either without any field applied to the underlying PEDOT:PSS substrate or with an electric field applied between contacts 1 and 3 (voltage of + 0.5 V or − 0.5 V) (see Fig. 1a), which are under the PEDOT:PSS contacting the electrolyte, or between the two outer contacts (+ 1 V or  − 1 V). We note that, during the cell culture studies, the voltage was applied just at the container borders, outside the electrolyte, and was limited to 1 V. This limiting voltage was intended to avoid water electrolysis^14,15^.

The EDL potential takes some time to stabilise and we find a typical value of ca. 0.3 V when a KCl (0.1 M) electrolyte is used. The variability of the OCP of PEDOT:PSS/electrolyte may be associated to the swelling of the polymer and diffusion of ions across the interface. After stabilization, the EDL potential is measured over time following the external bias application. As shown in Fig. 1b,c, when a bias of + 0.5 V is applied between contacts 1 and 3, the EDL potential at position 1 increases by ca. 0.27 V while that in position 3 decreases by ca. 0.23 V. The overall variation is around 0.5 V, equal to the bias voltage applied. It should be mentioned that the variation of the EDL potentials at contacts 1 and 3 should be equal in absolute value. The difference of ca. 40 mV, between 0.27 V and 0.23 V, is attributed to the slow dynamics of the system. A closer look at the shape of the EDL response (Fig. 1b,c) reveals an initial sharp variation, followed by a smoother evolution towards equilibrium. The complexity of the PEDOT:PSS system, a blend with phase separated domains and with different interactions with water (PEDOT^+^ is hydrophobic, whereas PSS^-^ is hydrophilic), combined with the above-mentioned slow evolution of the EDL potential towards equilibrium values, may explain the asymmetry in the EDL response at the two contacts. I find that if a negative bias is applied, the EDL potential varies in a symmetric way to the variation shown in Fig. 1. If the EDL potential is measured at the position 2, a very small variation of OCP (ca. 2 mV) for the same bias of 0.5 V applied between contacts 1 and 3 is measured. The observation of this very small variation is attributed to a small off-centre position of the ITO stripe 2, suggesting that it would be zero if the position was precisely half-way between contacts 1 and 3.

The variation of the EDL potential is very steep, taking place within a time interval of ca. 10 ms, which could be limited by the limiting reading speed of OCP (10 ms is the lower limit of the time interval between readings). As the bias potential is applied with an independent voltage source meter, we estimate that the EDL response takes place in less than 50 ms after the bias application.

When a 1 V bias is applied between the two outer contacts [0 and 4 in Fig. 1a)], away from the electrolyte container, the variation of the EDL potential follows the same pattern, increasing by 0.19 V at position 1, for instance, as shown in Fig. SI.1.

If PBS is used instead of the KCl electrolyte, a similar response of the EDL potential is observed (see Fig. SI.4), evidencing that the effect is present when the PEDOT:PSS surface contacts biological fluids (electrolytes).

**Figure 2:**
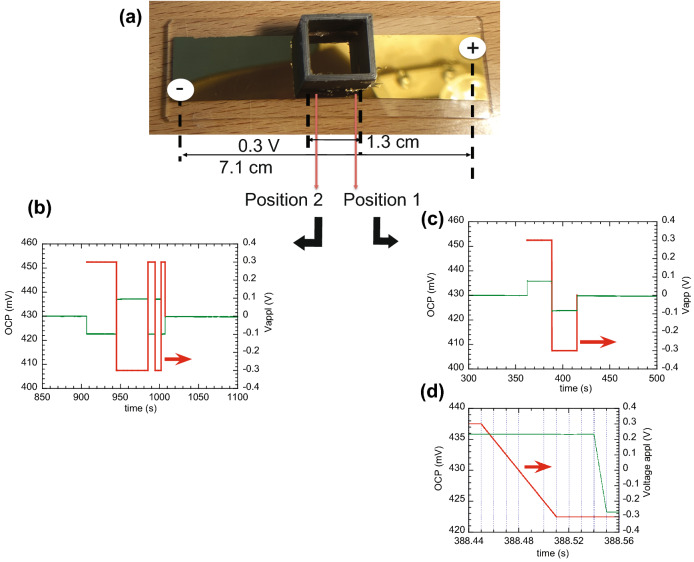
(**a**) Photograph of the electrolyte container glued to a gold substrate prepared by thermal evaporation on a glass slide, indicating the approximate positions of the leads used to apply the bias voltage (0.3 V) and of the probe lead (working electrode position) (positions 1 or 2); (**b**) and (**c**) variation of the EDL potential at positions 2 and 1, respectively, upon application of a bias (red), evidencing both the reverse variation of the EDL potential at the two positions for the same bias and its reversibility; (**d**) enlarged representation of the EDL potential variation of (**b**) when the bias changes from + 0.3 V to − 0.3 V, evidencing a delay between 90 and 40 ms in the EDL response, which is likely limited by the setup used.

A similar study was carried out using a gold substrate thermally deposited on a glass slide and KCl (aq., 0.1 M) as electrolyte. Due to the high conductivity of gold, I could not find a way to spatially localise the EDL under study. Two measuring positions were selected at opposite sides of the electrolyte container, as shown in Fig. 2. A similar fast modulation of the EDL potential is observed upon application of a bias of 0.3 V to the gold substrate. It should be mentioned, however, that, while for gold, the variation of EDL potential is a single step (within 10 ms) that of PEDOT:PSS takes longer to stabilise, usually longer than 30 ms (see Fig. SI.1).

The above results show that the electric field applied along the substrate (gold or PEDOT:PSS) induces a change of the EDL potential, which increases in a region close to positive potential and decreases near the negative potential. Due to the high conductivity of the substrates, the electric field should vary linearly along the substrates, this explaining the nearly zero effect on the EDL potential at the central position.

Electrochemical impedance spectroscopy (EIS) characterisation of the interface between PEDOT:PSS and the KCl electrolyte was made, without and with bias applied to the PEDOT:PSS film. The aim was to obtain information about the capacitance associated to the EDL, as capacity measurements have been widely used for EDL characterization. However, no clear effect of the bias was observed, as shown in Fig. SI.3. The measurements, within the maximum frequency range (1 MHz to 1 mHz), usually took ca. 80 min and it is found that the OCP at the end of the measurement is nearly zero (or a few mV). It appears that the measurement itself, involving an ac probing voltage of 0.01 V, affects the interface. This is an unexpected result in view of the large number of reported studies of the PEDOT:PSS/solution interface^18,19^.

Various models have been proposed to describe charge distribution in an EDL, in particular in the electrolyte (Ref 20 and references therein). In a metal/electrolyte interface, there will be an excess charge density at the metal surface, σ_M_, that is considered uniform, which is compensated by an excess ionic charge density (of opposite sign), on the electrolyte side, σ_E_, so that σ_M_ = − σ_E_. The models describing the organization of the ions on the electrolyte side have evolved along the years, starting with the simple Helmholtz model, where the EDL was modelled by a parallel-plate capacitor. The latest development was due to Grahame, that divided the ion distribution in three regions. However, a previous model of Stern, that considers an inner compact (Stern) layer and a diffuse layer, is sometimes used to model the capacitance of the EDL. It is worth mentioning that the diffuse layer extends up to the Debye length (*l*_D_), whose value is predicted to decrease with increasing ionic strength of the electrolyte. For a NaCl(aq, 0.1 M) electrolyte *l*_D_ is around 1 nm^20^.

The excess surface charge density on a planar metal (σ_M_) is related to the corresponding surface potential (ψ_0_) by the Grahame Eq. ^21^

1 $$\sigma_{M} = \sqrt {\left( {8n\varepsilon_{r} \varepsilon_{0} kT} \right) }\, sinh\left( {\frac{{ze\psi_{0} }}{2kT}} \right)$$

where ε_r_, ε_0_ and *n* are the relative permittivity, permittivity in vacuum and electrolyte concentration in bulk solution, respectively. Equation (1) shows that a higher surface potential originates a higher charge density (both σ_M_ and σ_E_ = σ_M_). Based on this equation, I consider that the application of a bias along the gold surface, creating an electric field and therefore a variation of the surface potential, induces a change of the excess surface density and, therefore, a variation of the potential associated to the EDL. This reasoning is likely applied to the highly conductive PEDOT:PSS, even though PEDOT:PSS is a blend of two polymers, which are phase separated and is expected to originate a quite complex interface with the electrolyte. It was found that PEDOT:PSS in contact with an electrolyte shows a very large, non-geometric, capacitance, which has been attributed to either a double layer between PSS^−^ and cations that enter the film from solution upon exchange with the PEDOT holes^22,23^ or to the internal electrical double layer formed at the interface between PEDOT^+^-rich and PSS^–^ rich domains^24,25^. The measured OCP is related to the EDL formed at the PEDOT:PSS blend/electrolyte interface and is possibly affected by the internal EDL^25^. The ability to modulate the EDL at the interface with an electrolyte, here reported, adds to the fascinating characteristics of this material.

To summarise, the new finding that the EDL potential is modulated by the application of an electric field in the conductive substrate, either gold or PEDOT:PSS, contacting an electrolyte, opens up new opportunities in terms of the development of existing and new devices in electronics and bioelectronics, and is likely to motivate the revisiting of the existing models describing their operation.

### Methods

The electrolyte container was prepared by 3D printing and the cross-linked PEDOT:PSS film, ca. 100 nm thick, was prepared as described previously^14,15^, using PEDOT:PSS CLEVIOS P AI 4083, from Heraeus, and 3-glycidyloxypropyl)trimethoxysilane (GOPS, Aldrich) as cross-linker. Silver and gold films were thermally evaporated on glass slides or glass slides with ITO stripes (after etching from ITO/glass substrates with diluted hydrogen chloride) in a vacuum evaporator (Edwards 306A). The electrolyte solutions were prepared with deionised water and either KCl, AuCl_3_ or AgNO_3_ (all from Aldrich). PBS (pH 7.4) was obtained from Aldrich. OCP and EIS measurements were carried out with a PalmSens4 system. The Ag/AgCl (3 M) reference electrode was obtained from Aldrich. The bias voltage was applied with a Keithley 2400 SourceMeter unit.

## Supplementary Information

Supplementary Information.

## References

[1] Parsons R (1990). Electrical double layer: Recent experimental and theoretical developments. Chem. Rev..

[2] Lyklema J (1964). The measurement and interpretation of electric potentials from a physico-chemical point of view. Med. Electron. Biol. Engng..

[3] Shirakawa, H., Louis, E. J., MacDiarmid, A.G., Chiang, C. K. & Heeger, A. Synthesis of electrically conducting organic polymers: Halogen derivatives of polyacetylene, (CH)_x_. *J. Chem. Soc. Chem. Commun.* 578–580 10.1039/C39770000578 (1977).

[4] Kim SH (2013). Electrolyte-gated transistors for organic and printed electronics. Adv. Mater..

[5] Rivnay J, Owens RM, Malliaras GG (2014). The rise of organic bioelectronics. Chem. Mater..

[6] Wallace GG, Moulton SE, Clark GM (2009). Electrode-cellular interface. Science.

[7] Venkatraman S, Hendricks J, King ZA, Sereno AJ, Richardson-Burns S, Martin D, Carmena JM (2011). In vitro and in vivo evaluation of PEDOT microelectrodes for neural stimulation and recording. IEEE Trans. Neural Syst. Rehabil. Eng..

[8] Sessolo M (2013). Easy-to-fabricate conducting polymer microelectrode arrays. Adv. Mater..

[9] Green R, Abidian MR (2015). Conducting polymers for neural prosthetic and neural interface applications. Adv. Mater..

[10] Khodaghloy D (2013). *In vivo* recordings of brain activity using organic transistors. Nat. Commun..

[11] Ghasemi-Mobarakeh L (2009). Electrical stimulation of nerve cells using conductive nanofibrous scaffolds for nerve tissue engineering. Tissue Eng. Part A.

[12] Thompson BC (2010). Conducting polymers, dual neurotrophins and pulsed electrical stimulation—Dramatic effects on neurite outgrowth. J. Control. Release.

[13] Martin DC, Malliaras GG (2016). Interfacing electronic and ionic charge transport in bioelectronics. ChemEletroChem.

[14] Pires F, Ferreira Q, Rodrigues CA, Morgado J, Ferreira FC (2015). Neural stem cell differentiation by electrical stimulation using a cross-linked PEDOT substrate: Expanding the use of biocompatible conjugated conductive polymers for neural tissue engineering. Biochim. Biophys. Acta.

[15] Sordini L (2021). Effect of electrical stimulation conditions on neural stem cells differentiation on cross-linked PEDOT:PSS films. Front. Bioeng. Biotechnol..

[16] Garrudo FFF (2021). Electrical stimulation on neural-differentiating iPSCs on novel coaxial electroconductive nanofibers. Biomater. Sci..

[17] Thrivikraman G, Boda SK, Basu B (2018). Unraveling the mechanistic effects of electric field stimulation towards directing stem cell fate and function: A tissue engineering perspective. Biomaterials.

[18] Koutsouras DA (2017). Impedance spectroscopy of spin-cast and electrochemically deposited PEDOT:PSS films on microfabricated electrodes with various areas. ChemElectroChem.

[19] Inácio PMC (2020). Ultra-low noise PEDOT:PSS electrodes on bacterial cellulose: A sensor to access bioelectrical signals in non-electrogenic cells. Org. Elect..

[20] Khademi M, Barz DPJ (2020). Structure of the electrical double layer revisited: Electrode capacitance in aqueous solutions. Langmuir.

[21] Chu C-H (2017). Beyond the Debye length in high ionic strength solution: Direct protein detection with field-effect transistors (FETs) in human serum. Sci. Rep..

[22] Rivnay J (2015). High-performance transistors for bioelectronics through tuning of channel thickness. Sci. Adv..

[23] Proctor CM, Rivnay J, Malliaras GG (2016). Understanding volumetric capacitance in conducting polymers. J. Polym. Sci. Part B Polym. Phys..

[24] Volkov (2017). Understanding the capacitance of PEDOT:PSS. Adv. Funct. Mater..

[25] Tybrandt K, Zozoulenko IV, Berggren M (2017). Chemical potential-electric double layer coupling in conjugated polymer-polyelectrolyte blends. Sci. Adv..

