# Supplementary material for: Modulation of the electrical double layer in metals and conducting polymers
Source: Sci Rep. 2022 Jan 10;12:307. doi: 10.1038/s41598-021-03948-8 (PMC8748889; doi:10.1038/s41598-021-03948-8)
Supplement: Supplementary file 1 — Supplementary Information. [file 41598_2021_3948_MOESM1_ESM.pdf]

Supporting Information

Modulation of the electrical double layer in metals and conducting polymers

Jorge Morgado

Instituto de Telecomunicações and Department of Bioengineering, Instituto Superior Técnico, Universidade de Lisboa, Av. Rovisco Pais, P-1049-001 Lisboa, Portugal

e-mail: jorge.morgado@lx.it.pt, jmfmorgado@tecnico.ulisboa.pt

The electrolyte container was prepared by 3D printing and the cross-linked PEDOT:PSS film, ca. 100nm thick, was prepared as described previously[1], using PEDOT:PSS CLEVIOS P AI 4083, from Heraeus, and the cross-linker (3-glycidyloxypropyl)trimethoxysilane (GOPS, Aldrich). Silver and gold films were thermally evaporated on glass slides or glass slides with ITO stripes (after etching from ITO-coated glass substrates with diluted hydrogen chloride) on a vacuum evaporator (Edwards 306A). The electrolyte solutions were prepared with deionised water and KCl, $AuCl_3$ or $AgNO_3$ (all from Aldrich). PBS was obtained from Aldrich. OCP and EIS measurements were carried out with a PalmSens4 system. The Ag/AgCl(3 M) reference electrode was obtained from Aldrich. The bias voltage was applied with a Keithley 2400 SourceMeter unit.

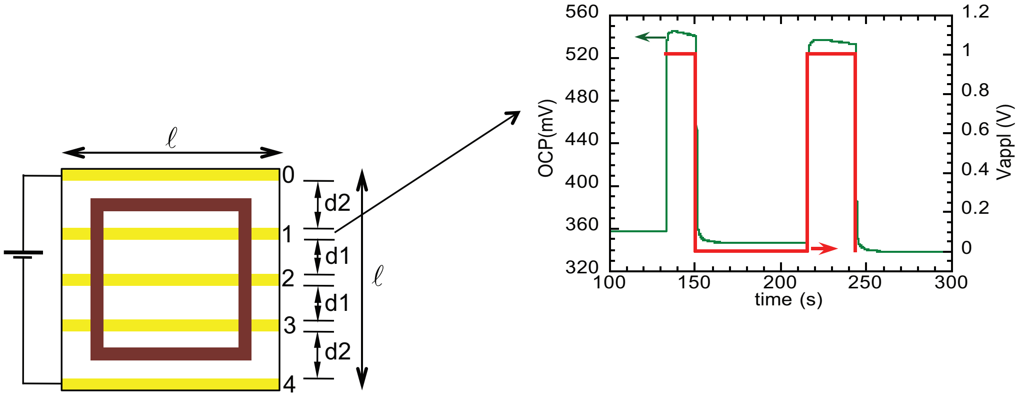

Figure SI.1. Modulation of the EDL potential at position 1 upon application of a bias of 1V between contacts 0 and 4.

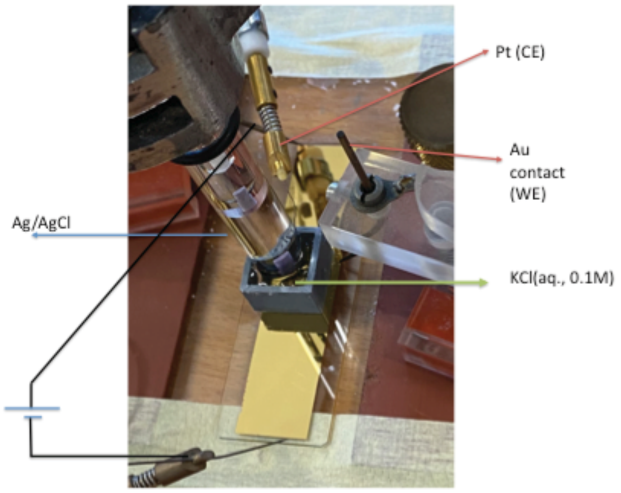

Figure SI.2. Photograph of the measuring setup, when characterising the gold/KCl interface.

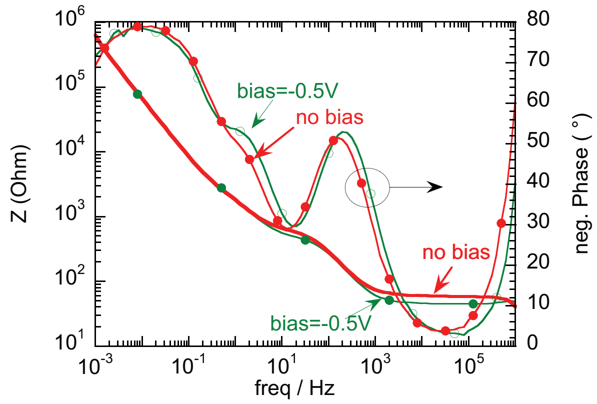

Figure SI.3. Impedance of the PEDOT:PSS/KCl interface (Bode plot) without and with a bias of -0.5 V applied to the PEDOT:PSS film (via an underlying ITO stripe). The measurements were made with a PalmSens4 system, at 91 frequencies in the frequency range 1 mHz-1 MHz, using an ac voltage of 0.01 V and an equilibration time of 4 s.

OCP was also used to measure the standard reduction potentials of Au^{3+}/Au and Ag^{+}/Ag electrochemical pairs. Setups similar to those shown in Figure 2 were used. Gold and silver (ca. 100 nm thick) were thermally evaporated over glass slides, the container was glued on top and, ca. 30 minutes later (to allow the glue to dry), a solution of $AuCl_3$ (aq, 0.05 M) or $AgNO_3$(aq, 1 M) was placed inside. The OCP was measured using the three-electrode arrangement discussed above. After stabilisation, we obtained an OCP of 0.41 V and 0.42 V for Ag^{+}/Ag. As these values refer to the Ag/AgCl reference electrode, whose standard electrode potential lies ca. 0.22 V above the potential of the standard hydrogen electrode (SHE)[2] we

estimate the standard reduction potential of the pair Ag^{+}/Ag as +0.63 V and +0.64 V, which is slightly lower than the tabulated value[3] of +0.7996 V. In the case of gold, we measured OCP values of 1.04 V and 1.06 V, which, after correction for the 1M concentration (standard condition) using Nernst equation, considering a temperature of 298 K,

$$E(V) = E^{0}(V) + \frac{0.0591}{3}\log\left[Au^{3+}\right] \quad (1)$$

translates into a value of the standard potential of 1.07 V and 1.09 V, respectively, for the Au^{3+}/Au pair with respect to the Ag/AgCl reference electrode. These values are translated into the standard reduction potentials for the pair with respect to the SHE of +1.29 V and +1.31 V, which are close to the tabulated value (+1.498 V)[3]. We believe the difference (ca. 0.2 V) between measured and tabulated values of the standard redox potentials can be due to the characteristics of the metallic films (namely, their crystallinity) and some atmospheric oxidation during the films' exposure to air during the 30 min required for the glue to dry (fixing the container on the films), before adding the electrolyte.

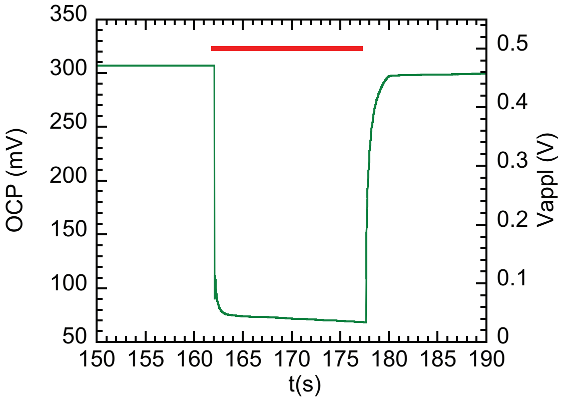

Figure SI.4 Modulation of the EDL formed at the PEDOT:PSS/PBS interface. The setup is similar to that shown in Figure 1. The bias pulse of +0.5 V, shown in red, was applied between contacts 1 and 3 and the OCP measurement was performed at contact 3. The modulation of the OPC (in green) is similar to that observed at the PEDOT:PSS/KCl(0.1 M) interface shown in Figure 1.

References

[1]Sordini L., *et al.* Effect of electrical stimulation conditions on neural stem cells differentiation on cross-linked PEDOT:PSS films. *Front. Bioeng. Biotechnol.* **9**, 591838 (2021).

[2]Bates, R.G. & MacAskill, J.B. Standard Potential of the Silver-Silver Chloride Electrode. *Pure&Applied Chem.* **50**, 1701-1706 (1978).

[3]CRC Handbook of Chemistry and Physics, 89th Edition, David L. Lide (Ed.), CRC Press (2008), page 8-20
